# Supplementary material for: Identification of Viruses Infecting Phalaenopsis Orchids Using Nanopore Sequencing and Development of an RT-RPA-CRISPR/Cas12a for Rapid Visual Detection of Nerine Latent Virus
Source: Int J Mol Sci. 2024 Feb 25;25(5):2666. doi: 10.3390/ijms25052666 (PMC10931582; doi:10.3390/ijms25052666)
Supplement: Supplementary file 1 [file ijms-25-02666-s001.zip › Supplementary Table S1_Revised.pdf]

Supplementary Table S1. Information on primer sets used in this study

Assay	Name	Sequence 5'-3'	Amplicon size	References
RT-RPA	NeLV-F1	CCTTCCTCGACCGCAGTGGCGACTTGCACA	105 bp	This study
	NeLV-R1	CCAAACTATTGGTGCTATTGATTCAGGCGG		
CRISPR/Cas12a	NeLV-crRNA	CCAATCCTTGCGAATTTAGCTATCTACACTTAGTAGAAATTACCCTAT AGTGAGTCGTATTAATTTC		This study
	ncrRNA-1	UAAUUUCUACUAAGUGUAGAUUCAGGUAAGUUAGGAUGCUUCCC		
	ncrRNA-2	UAAUUUCUACUAAGUGUAGAUGGGUAGAACUAACGAUGCCCUUU		
	ncrRNA-3	UAAUUUCUACUAAGUGUAGAUUCAGGCAAAUCCACUUCAACA		
	T7-crRNA-F	GAAATTAATACGACTCACTATAGGG		[1]
	ssDNA Reporter	6-FAM-TTATTCCCCC-BHQ-1		[2]
RT-PCR	CymMVK-F	ACAATAATTTGAAATAATCATGGGA	716 bp	[3]
	CymMVK-R	AAAACCACACGCCTTATTAAGTTTG		
	NeLV-F	CCTATGGATGAGATTAGRCCG	612 bp	[4]
	NeLV-R	GCAACCATACAGCCCACC		
	ORSVK-F	ACGCACAATCTGATTCGTATTGAA	528 bp	[3]
	ORSVK-R	TATCAACGTTATTTTCCTAAATAT		
	Orchid- ACT-F1	ACAGGTATTGTGCTTGATTCTGG	356 bp	[5]
	Phal-ACT4-R	ATCATGGATGGTTGGAAAAGGAC		

References

1. Srivastava, A.; Gupta, T.; Srivastava, S.; Dhir, S.; Kumar, P.; Singhal, T.; Rani, A.; Rishi, N. Development of a new Collateral Cleavage-independent CRISPR/Cas12a based easy detection system for plant viruses. *J. Virol. Methods* **2022**, *300*, 114432. https://doi.org/10.1016/j.jviromet.2021.114432
2. Lee, S.; Nam, D.; Park, J.S.; Kim, S.; Lee, E.S.; Cha, B.S.; Park, K.S. Highly efficient DNA reporter for CRISPR/Cas12a-based specific and sensitive biosensor. *Biochip J.* **2022**, *16*, 463-470. https://doi.org/10.1007/s13206-022-00081-0
3. Chung, B.-N.; Yoon, J.-Y.; Kim, M.-S. Viral infection of tissue cultured orchids and evaluation of damages. *Plant Pathol. J.* **2010**, *26*, 194-197. https://doi.org/10.5423/PPJ.2010.26.2.194
4. Heo, E.S.; Choi, S.H. Nerine latent virus diagnosed in *Hippeastrum* bulbs in Korea. *Hortic. Sci. Technol.* **2021**, *39*, 507-513. https://doi.org/10.7235/HORT.20210045
5. Jeong, D.-S.; Hong, K.H.; Park, K.-I. Detection of Cymbidium mosaic virus and Odontoglossum ringspot virus in Phalaenopsis in Korea. *Hortic. Sci. Technol.* **2021**, *39*, 514-520. https://doi.org/10.7235/HORT.20210046
