# Supplementary figures and images for: Identification of Viruses Infecting Phalaenopsis Orchids Using Nanopore Sequencing and Development of an RT-RPA-CRISPR/Cas12a for Rapid Visual Detection of Nerine Latent Virus
Source: Int J Mol Sci. 2024 Feb 25;25(5):2666. doi: 10.3390/ijms25052666 (PMC10931582; doi:10.3390/ijms25052666)

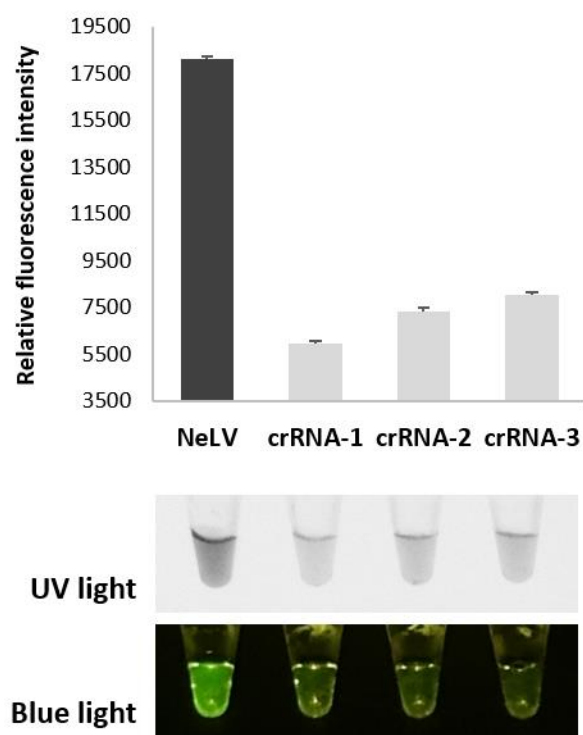

Figure S1: Confirmation of specificity with nonsense crRNA control

Supplement: Supplementary file 1 [file ijms-25-02666-s001.zip › Figure S1.pdf]
